# Road to Acquisition: Preparing a MEMS Microphone Array for Measurement of Fuselage Surface Pressure Fluctuations

**DOI:** 10.3390/mi12080961

**Published:** 2021-08-14

**Authors:** Thomas Ahlefeldt, Stefan Haxter, Carsten Spehr, Daniel Ernst, Tobias Kleindienst

**Affiliations:** German Aerospace Center (DLR), Bunsenstr. 10, D-37073 Göttingen, Germany; stefan.haxter@dlr.de (S.H.); carsten.spehr@dlr.de (C.S.); daniel.ernst@dlr.de (D.E.); tobias.kleindienst@dlr.de (T.K.)

**Keywords:** microphone array, turbulent boundary layer, Kapton foil, flight test, wave number decomposition, propeller, spatial resolution, flexible circuit board

## Abstract

Preparing and pre-testing experimental setups for flight tests is a lengthy but necessary task. One part of this preparation is comparing newly available measurement technology with proven setups. In our case, we wanted to compare acoustic Micro-Electro-Mechanical Systems (MEMS) to large and proven surface-mounted condenser microphones. The task started with the comparison of spectra in low-speed wind tunnel environments. After successful completion, the challenge was increased to similar comparisons in a transonic wind tunnel. The final goal of performing in-flight measurements on the outside fuselage of a twin-engine turboprop aircraft was eventually achieved using a slim array of 45 MEMS microphones with additional large microphones installed on the same carrier to drawn on for comparison. Finally, the array arrangement of MEMS microphones allowed for a complex study of fuselage surface pressure fluctuations in the wavenumber domain. The study indicates that MEMS microphones are an inexpensive alternative to conventional microphones with increased potential for spatially high-resolved measurements even at challenging experimental conditions during flight tests.

## 1. Introduction

The characteristics of pressure fluctuations on the airplane fuselage govern the vibro-acoustic excitation of surface panels exposed to the boundary layer flow. These pressure fluctuations are caused either by the Turbulent Boundary Layer (TBL) itself or acoustic fluctuations induced by-for instance-the engine or by airframe noise. Experimental data of these pressure fluctuations are difficult to obtain in ground-based tests due to the high Reynolds numbers required. Flight tests are an appropriate method to obtain knowledge about the characteristics of these pressure fluctuations under realistic conditions.

Sensors suitable for this task require a high overload point to resolve the high-amplitude pressure peaks within the turbulent boundary layer. The size of the sensor influences the spatial resolution of the measurement, i.e. the size of detectable pressure structures and the measurable coherence between any two sensors. Surface microphones that are currently commercially available and for in-flight testing are comparably large and thus focus on providing acoustic pressure fluctuations. Small sensors suitable for capturing both acoustic and hydrodynamic pressure fluctuations are to our knowledge not very common.

This paper aims to present the experience gained from the design, application, and evaluation of a flight test with a phased microphone array to measure the fuselage excitation by the acoustic as well as hydrodynamic wavelength. The array developed in-house for this task consists of MEMS pressure sensors, concealed beneath a protective layer.

Commercially available surface microphones typically have a sensitive surface size between 1/2 inch and 1/4 inch and a dynamic range reaching up to 160–175 dB. These microphones are reasonably thin (thickness between 1.4 mm to 2.5 mm) so that they do not excessively protrude into the TBL during the measurement. They are suitable for the measurement of the acoustic excitation of an aircraft fuselage by (acoustic) wavelengths larger than the sensitive surface size (acoustic wavelengths of frequencies up to 20.00 kHz).

The measurement of small, high-energy TBL structures connected to fuselage excitation is a complex task compared to the assessment of acoustic pressure fluctuations. In the acoustically relevant frequency range, the wavelengths of typical hydrodynamic pressure fluctuations are small compared to acoustic wavelengths [1]. Thus, the acquisition of data in the flight test scenarios requires prior knowledge of the measurement environment and a careful design of the MEMS array.

The discourse in this paper will go as follows: after a brief introduction to the state of the art, the challenges of measuring in a flow using sensors of different sizes are described in Section 2. The in-house MEMS pressure sensor array is introduced in Section 3 followed by the ground tests conducted with and without flow (Section 4). The setup for the final in-flight measurements is presented in Section 5 including selected results demonstrating the feasibility and performance of the introduced MEMS microphone array.

### State of the Art

Currently, the state-of-the-art sensors for measuring these fluctuating pressure distributions are small (pinhole-mounted) piezoresistive pressure transducers (e.g., Kulite) as shown for in-flight measurements [2,3]. These sensors typically are rather expensive. Moreover, using this type of sensor has several drawbacks. The inherent noise level may mask the lower level acoustic excitation, which can nevertheless contribute to the fuselage excitation due to its large spatial coherence. The space required for mounting the sensors limits possible locations for measurements on the fuselage mostly to passenger windows replaced by dummy windows. Furthermore, the mounting condition of the sensors governs the achievable results by possible flow-induced self-noise, pressure damping within a sound hole, or the minimum distance between two sensors (see Section 2).

Alternatives to these pressure sensors are MEMS microphones due to their small size and low cost. In aeroacoustic testing, one must distinguish between in-flow sensors, which are mounted on any kind of surface that is exposed to the flow-field, and out-of-flow sensors, which are mounted out of the flow-field of an open jet wind tunnel. A recent example with out-of-flow sensors by Zhou et al. [4] shows the application of a 256 MEMS microphone array in an open jet wind tunnel for aeroacoustic measurements. In the current paper, only the in-flow placement of acoustic sensors and their unique problems are discussed.

In 1999 Sheplak et al. [5] developed the first custom aeroacoustic MEMS microphone. They also defined the critical performance parameters of an aeroacoustic MEMS microphone which state, that the dynamic range should be 60 dB to 160 dB and the upper frequency limit should be higher than 50 kHz. The resulting sensor implementation offered an upper frequency limit of 6 kHz (predicted 300 kHz) with a noise floor of the packaged sensor of 92 dB/$\sqrt{\mathrm{Hz}}$ (predicted 63 dB/$\sqrt{\mathrm{Hz}}$). The maximum sound pressure level was 155 dB.

Several publications on custom aeroacoustic MEMS microphones were published since then (see Table 8-2 in [6]). A good summary of these developments and details on the fabrication process are the Ph.D. theses of Williams [7] and Reagan [6]. Both authors developed new custom MEMS microphones which are meeting the requirements stated above. However, these sensors are not available on the open market. Therefore, implementations of MEMS microphones in experimental acoustic setups are based on commercially available sensors.

In comparison to surface microphones, commercially available MEMS microphones have a limited Acoustic Overload Point (AOP) of 135 dB maximum. While being sufficient for the consumer market, the small-scale hydrodynamic excitation within the TBL can exceed this limit already at moderate flow speeds of 30–50 m/s (see Section 4.2.1) and overload the sensors. Microphones in general show a nonlinear attenuation of the output amplitude response once the input amplitude has an order of magnitude similar to the AOP. Depending on how this compression of the signal is implemented in the sensor for very high input amplitudes, an undetectable and irrecoverable shaping of the signal, a high nonlinear distortion or sensor malfunctioning may occur.

Shams et al. [8] developed a 128 MEMS microphone array for aeroacoustic testing in 2004 and successful aeroacoustic measurements in a wind tunnel were first conducted by Humphreys et al. [9] in 2005 using MEMS microphones from Knowles. Both authors do not mention a problem with a low acoustic overload point. Sanders et al. [10] embedded digital MEMS microphones in trailing-edge serrations in order to measure the hydrodynamic coherence length. They state that MEMS microphones with a higher acoustic overload point should be used for Reynolds numbers larger than $7\times 10^{5}$ due to problems with signal clipping. Leclere et al. [11] and Salze et al. [12] used a MEMS microphone array for turbulent boundary layer measurements in a wind tunnel and turbofan duct. Both do not report any type of acoustic overload with measurements conducted at speeds from 0 m/s to 50 m/s free stream velocity.

To overcome the limitations of the low overload point, the MEMS microphones in the current study were covered with a Kapton foil of thickness 25 μm to attenuate the pressure excitation by approx. 38 dB. Covering the sensors has the positive side-effect of protecting the MEMS sensors on the fuselage from humidity while at the same time smoothing the surface by covering surface irregularities in the vicinity of the sensor (e.g., pinhole edges). The response of this Kapton foil to acoustic waves must be considered in the analysis. Calibration measurements of the foil are introduced in Section 4.

## 2. Sensors in Flow

When measuring pressure fluctuations underneath a turbulent boundary layer, the ratio of the size of the sensitive microphone surface relative to the “size” of the turbulent structure in the boundary layer can affect the measurement output. More specifically, the wavenumber of the turbulent structure’s pressure fluctuation has to be small enough in order for the sensor to spatially resolve the fluctuations. If the sensor is not able to resolve the turbulent structure, signal attenuation can occur.

This was described by Schewe [1] who recorded signals from microphones of various sizes flush-mounted underneath a TBL. Schewe introduced a dimensionless sensor diameter $d^{+}=du_{\tau }/\nu$ dependent on the inner wall variables, such as the wall shear velocity $u_{\tau }$ and the kinematic viscosity ν. This dimensionless sensor diameter was used to describe the dependence of several higher-order moments of the the normalized total fluctuating pressure, including power $p_{\mathrm{rms}}/q_{\infty }$ and flatness $\bar{p^{4}}/p_{\mathrm{rms}}^{2}$.

The fluctuating power was found to decrease with dimensionless sensor size and is connected to rare high-power events, indicated by a similar increase in flatness of probability density function of the pressure fluctuations. The effect is quite significant: when using a sensor of $d^{+}\approx 300$, only half of the pressure power is received compared to the power estimated at an extrapolated value of $d^{+}=0$. The larger share of power measured with high-resolution sensors implies that a lot of fluctuating power is contained in the small-scale structures of the boundary layer. In addition to possibly underestimating the power content of the pressure fluctuations, the pressure applied by these small-scale structures may exceed the AOP of sensors used. Schewe found that the portion of fluctuation power not resolved by larger diameters was connected to higher frequencies of the fluctuations, thus linking the higher frequencies with higher wavenumbers.

The dampening of higher wavenumbers can be modeled by averaging a fluctuation over a sensitive surface of the desired shape. For circular and various other transducer shapes this was done by Ko [13] and will be shown below in Section 2.1. The wavenumber characteristics of TBL surface pressure fluctuations were described by Corcos [14] who formulated a spectral dampening compensation for power levels at higher frequencies. The effects of different pressure characteristics on the results are described in Section 2.2.

### 2.1. Sensitive Surface Averaging

**Figure 1:**
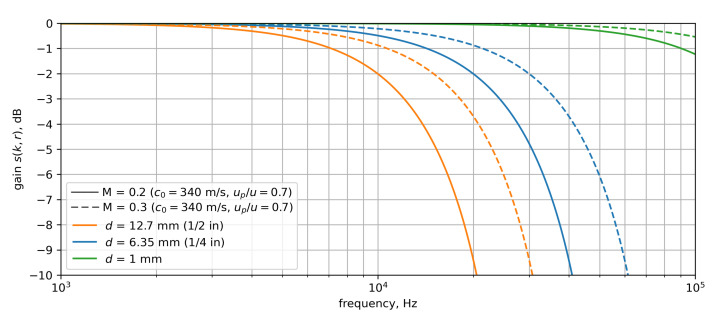
Modeled attenuation of hydrodynamic surface pressure fluctuations as a function of frequency at different Mach numbers and sensitive surface radii.

Concerning the characterization of signal attenuation as a function of the wavenumber, Ko [13] modeled the effect of a cosine-shaped surface pressure fluctuation over a circular shaped transducer. The resulting attenuation was found to be a function of the size ratio a=k·r of sensitive surface radius *r* and wavenumber *k* of the surface pressure fluctuations. The gain s(k,r), resulting from the modeled surface attenuation is given by

(1) $$s\left(k,r\right)=\frac{2J_{1}\left(k\cdot r\right)}{k\cdot r},$$

with $J_{1}\left(...\right)$ being the Bessel function of first kind of order one. The gain s(k,r) resulting from Equation (1) is shown in Figure 1 in dB for a range of frequencies with exemplary propagation velocities. For TBL convection, the phase propagation wavenumber $k_{p}$ is used for the relation of sensor size and wavenumber in Equation (1). It is obtained by using $k_{p}=f/u_{p}$ with $u_{p}$ being the phase velocity. The phase velocity describes the speed of signal phase changes over the array and takes values of approximately $u_{p}\approx 0.7u$, with *u* being the flow velocity determined from the Mach number $M=u/c_{0}$. Furthermore, *f* describes the frequency, and $c_{0}$ = 340 m/s the speed of sound. With constant phase velocity, wavenumber is proportional to the frequency and thus, the stronger the attenuation effect gets. For TBL pressure fluctuations propagating over the largest sensitive surface area (r=12.7 mm = 1/2 in) at a velocity of *M* = 0.2 the loss in gain does not exceed 1 dB below 10 kHz. At first glance, this appears to be sufficient for most acoustic full-scale tests with Helmholtz-number unity. However, TBL pressure fluctuations have a limited coherence length which pushes some of the wavenumber content of the convective propagation towards higher wavenumber values. This will be explained below.

### 2.2. Influence of Limited Coherence Length

**Figure 2:**
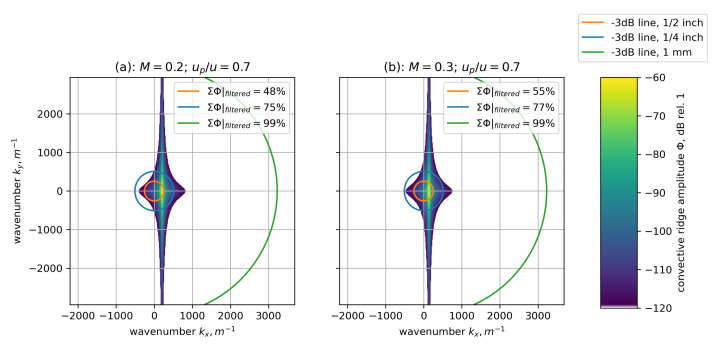
Exemplary attenuation caused by broadening of sources in the wavenumber domain due to limited coherence length. (**a**) *M* = 0.2 (**b**) *M* = 0.3.

While acoustic pressure fluctuations keep their self-similarity when propagating over large distances, hydrodynamic TBL pressure fluctuations do not. The hydrodynamic propagation mechanism has a limited coherence length-a characteristic length to describe the size of a pressure patch where pressure is applied to a surface coherently. Besides the lower propagation velocity of subsonic hydrodynamic pressure fluctuations, their limitation in coherence length enlarges the wavenumber content contained in the propagation mechanism and adds to the attenuation of spectral power. The effect was described by Corcos [14]. Figure 2 shows an exemplary hydrodynamic source modeled in the wavenumber domain after Graham [15] at a frequency of 10 kHz for two velocities *M* = 0.2 in Figure 2a and *M* = 0.3 in Figure 2b. The velocity fraction for convective transport was assumed again at 0.7. fraction At a frequency of 10 kHz, this results in convection wavenumbers of $k_{c}\approx$ 210 m${}^{-1}$ and $k_{c}\approx$ 140 m${}^{-1}$ respectively, where the center of the convective ridge is located. Using a set of limited coherence lengths of $l_{x}=$ 5 cm and $l_{y}=$ 5 mm on the sources leads them to spread out in $k_{x}$- and $k_{y}$-direction rather than being a point source located at $k_{c}$. Especially due to the shorter coherence length in *y*-direction, the convective ridge can be observed to be spread out much more prominently in $k_{y}$-direction, resulting in a much higher wavenumber content.

In Figure 2, the −3 dB B-thresholds of sensors sizes 1/2 in, 1/4 in, and 1 mm are shown as orange, blue and green, respectively. For the case of M = 0.2 the center of the convective source is still contained within the −3 dB-line of the 1/2 in sensor. However, due to the limited coherence length, the hydrodynamic source is spread out towards higher wavenumbers and less content is contained within the passband of the spatial filter of the sensitive surface. This leads to this wavenumber content being attenuated.

Raising the flow velocity to M = 0.3 shifts the center of the convective ridge closer to the origin of the plot. However due to the large spread of the convective ridge still only a fraction of the energy is picked up by the sensor. This applies as well for the 1/2 inch sensor with its −3 dB line shown in blue, but to a lesser extent. Using a sensor of size 1 mm will yield a much larger passband of the spatial filtering as shown by the green curve. Here, almost the entire energy contained in the convective ridge is picked up.

The amount of source content contained within the −3 dB-line for each of the two sensor sizes is shown in the legend of each figure segment as a percentage of the total energy contained in the simulated source. In this simplified exemplary case, a sensor having a diameter of 1/2 inch would only capture 48% of the total energy at M = 0.2 and 55% at M = 0.3. The simulated 1/4 inch microphone would yield 75% and 77% for the two Mach numbers, and the 1 mm sensor is able to recover 99% of the energy for both simulated velocities under consideration.

Small sensors are therefore required for characterizing the TBL surface pressure fluctuations. Apparently, the difference in measured signal level due to the sole influence of velocity and convective wavenumber has a minor impact compared to the impact of a small coherence length.

The use of the −3 dB-threshold in the above example was used exemplary for illustration of the passband of the spatial filter constituted by each sensor’s size. This is not to be seen as a hard cutoff, but rather as a smooth transition from passband to stopband as shown in Figure 1.

#### 2.2.1. Influence on Measurement Time

**Figure 3:**
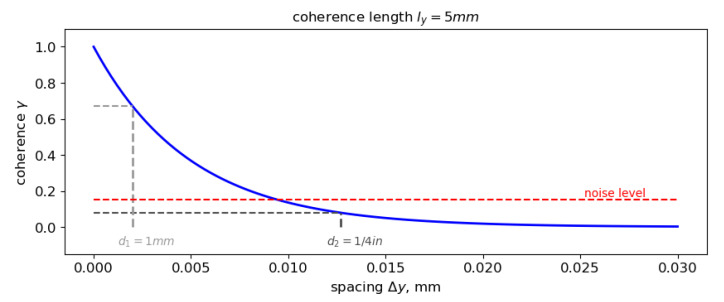
Different sensor sizes allow for different sensor spacings. The level of coherence between two signals from sensors spaced closer together is high, allowing for the determination of the coherence drop even at higher noise levels.

Besides having a smaller sensitive surface, smaller sensors can be placed closer together on a surface. This enables the measurement of smaller coherence lengths when measurement time is limited. A sketch of this effect is shown in Figure 3 where the signal coherence level is modeled [16] exemplary for small sensor separation distances in cross-flow direction Δy in the presence of a very small coherence length in cross-flow direction $l_{y}=5$ mm. $l_{y}$ takes very small values in the presence of thin boundary layers that may occur at the position of the flight deck in the front of the aircraft. The noise level is proportional to the inverse square root of the number of averages applied to the signal when evaluating and thus, very long measurement times are required to reduce the noise level. This can be circumvented to some extent by using sensors spaced closer together in a way that a higher signal coherence is still present and above the noise level even for shorter measurements. In order to better capture the proceedings underneath the boundary layer, in this region of the fuselage, the use of small sensors is advisable.

#### 2.2.2. Requirements for Sensors in Flow

Resulting from the aforementioned characteristics of sensors in flow, several requirements can be deduced in order to optimize an array for flow measurements.

- The sensor should be able to resolve small turbulent structures of high wavenumber. This requires a small sensor. Typically, the characteristic size of the sensor should be small compared to relevant scales in the TBL (e.g., the boundary layer thickness and inner length scales)
- Sensors should be placed closely together to allow for capturing hydrodynamic signal correlation even of small turbulent structures. This requirement calls for small sensors as well.
- The sensor should have a high dynamic range (not mentioned in the section above): Acoustic and hydrodynamic pressure fluctuations typically do not exhibit the same amplitude. Often, one of the two sources has a significantly higher amplitude than the other one. In order to resolve a source with low amplitude in the presence of a source with a very high amplitude, the dynamic range of the sensors needs to be large. This applies not only to combinations of hydrodynamic/acoustic sources but also to combinations of acoustic/acoustic sources and hydrodynamic/hydrodynamic sources.

## 3. MEMS Array Structure

In this section, the MEMS microphone array used for the flight test is introduced, presenting its general properties and structure as well as the microphones.

With the intention in mind to perform array measurements in parallel with analogue high-precision condenser measurement microphones for reference, analogue MEMS sensors of type ICS-40617 [17] were selected for the array. For time-synchronous acquisition, all analogue sensors were recorded by the same data acquisition system.

The MEMS microphone has dimensions of 3.50 mm by 2.65 mm with a thickness of 0.98 mm. The diameter of the sensor port and the diaphragm is 1 mm. It was mainly chosen for its reasonably high dynamic range of 111 dB and an AOP with 10% THD+N at 129 dB. However, for aeroacoustic measurements the AOP at 2% is of interest to gain reliable data for the evaluation. According to the data sheet [17], at 2% the AOP is approximately around 127 dB. The sensor also shows a nonlinear amplitude output response above the AOP, i.e., signal compression, as mentioned in Section 1.

**Figure 4:**
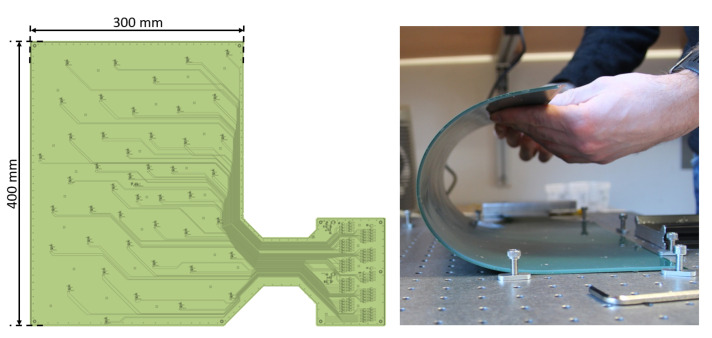
Left: Schematic of the printed circuit board with dimensions. Right: Bending demonstration of the array.

The MEMS microphone array was constructed on the basis of a flexible Printed Circuit Board (PCB) to enable an application onto curved surfaces. The PCB was manufactured in multilayer design using 6 layers with the two outer layers being used for shielding the signal lines on the inner layers. The selected dimensions for the aperture of the array were chosen to be 400 mm by 300 mm as depicted in Figure 4 (left side) with a thickness of the PCB of 0.45 mm. In combination with the thickness of the MEMS sensor a combined thickness of 1.45 mm was achieved.

The sensor port of the MEMS is located at the connector side which enabled an installation on the far side of the PCB with regard to the flow. Each sensor port was aligned with a through-hole of size 1 mm in the PCB connecting it to the smooth flow side of the array. For flush mounting on smooth surfaces, for insulating the electronic parts, and for moisture and dust protection during a test, the backside of the array (with the protruding sensor) was cast with silicone to an overall and equal thickness of the array of 1.55 mm. The finished MEMS microphone array on the flexible PCB responded well to bending as illustrated in Figure 4 (right side) without damaging the solder joints on the circuit board.

In total, 45 acoustic MEMS sensors were placed on the PCB to form the array with the focus set on demonstrating their applicability in flight testing in general. The MEMS microphones were arranged in a Vogel spiral suitable for acoustic beamforming [18].

**Figure 5:**
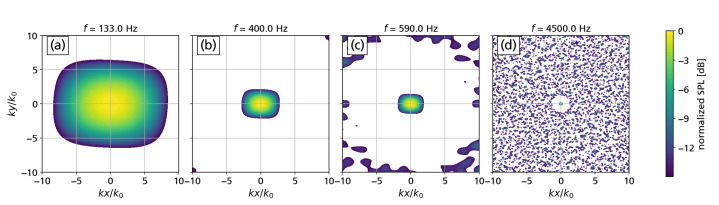
(**a**–**d**) Point spread function (PSF) of the array in wavenumber space at selected frequencies.

The point spread function for wavenumber space beamforming is shown in Figure 5 for selected frequencies.

As expected, a unity source in the center of the map exhibits a broad main lobe. The main lobe width decreases as the evaluation frequency increases. For a dynamic range of 15 dB some grating lobes start to appear at *f* = 590 Hz in the selected focus grid range of $-10\leq k_{x}/k_{0},k_{y}/k_{0}\leq 10$ approximately 9 dB below the maximum. At 4500 Hz, the width of the main lobe further decreases, yielding a higher resolution of the array. However, more grating lobes appear reaching levels of approximately 6 dB below the maximum. The analytical point spread functions are used for a DAMAS2.1 deconvolution attempt in Section 5.

The maximum difference in cable length was estimated to be the diagonal of the array area and thus 0.5 m. With an estimated delay caused by the cables of roughly 5 ns/m (corresponding to 0.67 times the speed of light), a maximum time delay of 2.5 ns is estimated between two channels of the array. This accounts for a maximum phase error of 0.09 deg at 100 kHz which is considered to be acceptable.

The MEMS required an electrical power supply voltage of 3 V which was provided by two batteries of type AA. The power was delivered to the array by utilizing two dedicated pins in the cable connectors.

### 3.1. Data Acquisition System

In this paper, two similar data acquisition systems were used. One system (“GBM Viper-48”) is capable of recording 48 channels up to 250 kHz at a bit depth of 16 bit, allowing for a maximum dynamic range of 96 dB.

The other system (“GBM Viper-HDR”) is capable of recording 64 channels up to 250 kHz at a bit depth of 24 bit, allowing for a maximum dynamic range of 144 dB.

Each channel of the two systems is equipped with its own sigma-delta A/D conversion unit and all A/D converters are synchronized and receive their clocking signal from one common clock source. Both systems possess a second-order high-pass filter with a cutoff frequency of 500 Hz which was used to reduce the influence of the low-frequency noise prior to A/D conversion and to make better use of the dynamic range of the system.

## 4. Preliminary Tests

The array and the sensors had to be examined extensively to ensure their functionality for the flight test. A total of four preliminary tests were performed. First, the in-situ frequency response of the individual microphones was determined without flow in an anechoic chamber. Second, a test was performed to study their behavior at low flow speeds. Observations made here lead to the introduction of Kapton foil as a treatment. Third, the influence of the foil on the frequency response was measured. Forth, a final test at comparable flight speeds in an industrial wind tunnel was performed in order to assess the influence of the foil and flow on the MEMS microphone’s frequency response compared to a reference sensor.

### 4.1. Frequency Response

The data sheet [17] lists a frequency response that is typical for this type of microphone, showing a flat response from 200 Hz to 10 kHz. Here, the individual frequency response of the MEMS sensors for a range up to 100 kHz was measured in the anechoic chamber of the aeroacoustic laboratory of DLR Göttingen. This was performed using two loudspeakers: a calibration speaker (B&K Omnisource 4295) for frequency range up to 6 kHz and a tweeter (ELAC-4Pi-II) for the frequency range from 5 kHz to 100 kHz. The MEMS microphone array was positioned at a 3.00 m distance to the speakers. A condenser microphone (B&K Typ 4944) with known frequency response was used as reference. This microphone was positioned in the center of a metal plate that had the dimensions of the MEMS microphone array, to mimic the influence of the array itself. In order to minimize the influence of source directionality especially of the tweeter special attention was paid to exact positioning when the MEMS array was exchanged for the reference plate. Here, the 24-bit GBM Viper-HDR data acquisition system was used (see Section 3.1).

The frequency response *H* was then calculated by comparing the reference microphone spectrum $G_{Y}$ with those of the MEMS array microphone spectra $G_{X}$:

(2) $$H\left(f\right)=\frac{G_{X}\left(f\right)}{G_{Y}\left(f\right)}$$

the measurement time was 30 s at a sampling frequency of 200 kHz. The data was then processed using an overlap of 50% and a fast Fourier transform block size of 5000 samples, with a Hann window, yielding 2399 averages and a narrowband frequency resolution of 40 Hz. The calculated results were then smoothed with a Savitzky-Golay filter [19] with a window length of 51 and a poly order of three.

**Figure 6:**
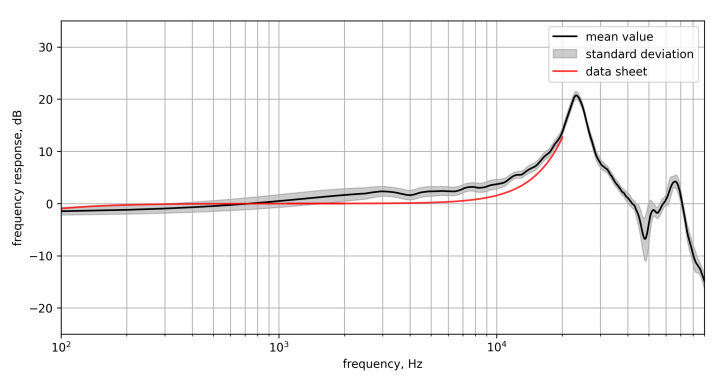
Mean frequency response of all array sensors with standard deviation in comparison to the data sheet.

Figure 6 shows the frequency response which was calculated by merging both results from the two separate measurements with different speakers and frequency range from 5 kHz to 10 kHz using a linear weighted transition. For the range of 300 Hz to 10 kHz the frequency response is approximately constant. For higher frequencies, a resonance peak is visible at 22 kHz and an anti-resonance drop at 44 kHz. The standard deviation of the frequency responses of all microphones is almost negligible with somewhat larger values from 500 Hz to 1 kHz and around the anti-resonance drop.

In general, the measured mean frequency response shows a low standard deviation between all MEMS sensors. The frequency response from the data sheet shows an almost perfect flat response from 10 Hz to 17 kHz. The comparison shows somewhat lower values of the measured frequency response below 1 kHz and somewhat higher values above 1 kHz. These deviations are probably due to inaccuracies in the measurement setup (influence of the measuring room and setup).

### 4.2. Tests in Flow Conditions

#### 4.2.1. First Tests (AKG)

The first flow application tests were performed in the aeroacoustic wind tunnel facility (AKG) of the aeroacoustic laboratory of DLR Göttingen. This small wind tunnel of Göttingen type has a 0.4 m by 0.4 m open test section with a length of approximately 2 m and can be operated at flow speeds up to *u* = 50 m/s.

**Figure 7:**
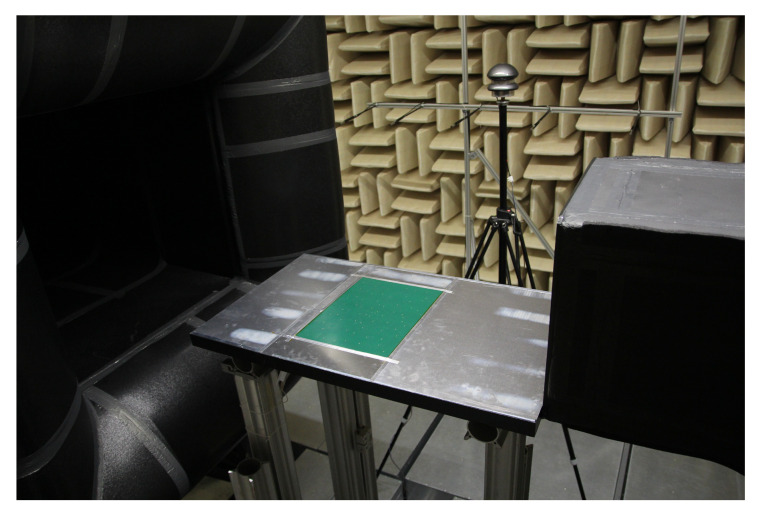
Photo of the measurement setup for the first tests under flow conditions. The MEMS array is mounted on the plate that is used as an extension of the bottom wind tunnel wall.

The microphone array was flush-mounted in a plate that was used as a extension of one wind tunnel wall with its surface aligned with and extending the bottom wall of the nozzle of the wind tunnel as shown in Figure 7. The tests were performed at different flow speeds ranging from *u* = 5 m/s to *u* = 40 m/s.

Here, the 24-bit GBM Viper-HDR data acquisition system was used (see Section 3.1). To lower the required dynamic range for recording the signals, which is mainly caused by the dominant low-frequency wind tunnel noise and hydrodynamic pressure, the 500 Hz high-pass filter was applied.

**Figure 8:**
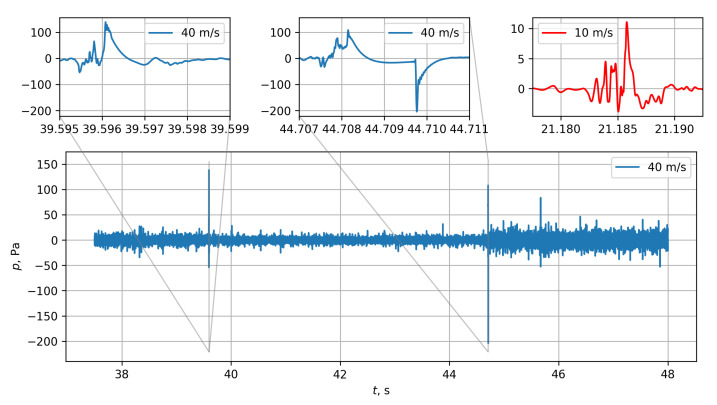
Time series of a chosen MEMS microphone at 40 m/s (blue) with zoomed-in sub-figures of peculiar events. For comparison, a zoomed-in result is shown at 10 m/s (red).

The individual MEMS measurements were investigated as time signals and spectra. While observing the live-feed from an ongoing measurement, it appeared that some online spectra would freeze and not change their shape even after turning off the wind tunnel. The observation was made at flow speeds from *u* = 20 m/s and above. Therefore, the time signals of the microphones were investigated in detail. Figure 8 shows a segment of a recorded time signal from an exemplary microphone at a flow speed of *u* = 40 m/s (blue).

Several isolated peaks are conspicuous, exceeding the predominant fluctuation amplitude by far. An interesting observation can be made after the second peak in Figure 8 at t≈ 44.7 s. The fluctuation intensity takes different levels before and after the peak, with the higher level continuing throughout the end of the graph. At a closer inspection after zooming in, the isolated peaks exhibit peculiar details. For the first event at t≈ 39.6 s, a quick increase of fluctuation energy can be observed leading up to a strong peak exceeding 140 Pa (corresponding to 137 dB). After the peak, an exponential decay of the signal can be observed, leading to an apparent regular signal again after one to two milliseconds. The second event shows a similar behavior, with the first exponential decay being followed by a steep and sudden drop (−200 Pa within 1 sample) in level and a subsequent second exponential mitigation.

The exponential decay after the high-amplitude events is caused by a clipping of the signals in the sensor and the signals being recorded with a 500 Hz high-pass filter applied to them.

Contrary to the first event, the overall fluctuation amplitude appears to be increased by a factor of two. The signal of this channel persisted to fluctuate even after turning off the wind tunnel. After a waiting time of several minutes, the sensor behavior returned to normal. This behavior occurred in approximately 10% of the sensors when subjected to flow velocities exceeding 20 m/s. Sensors exhibiting this behavior were located exclusively within the shear layer on the right and left sides of the array, produced by the corresponding nozzle edges. Both, the sensor behavior returning back to normal after some time, as well as the highly localized occurrence of the phenomenon in the experiment lead to the conclusion that it was not just a bad batch of sensors causing the observed behavior.

For comparison, a comparable signal from a sensor exhibiting the strange signal behavior is shown in Figure 8 for a lower flow velocity of *u* = 10 m/s (red). Although an event is visible exhibiting a similar fluctuation behavior leading up to a main peak, no exponential drop is observed after the main peak. For the case at *u* = 10 m/s, this type of event was found significantly more often at locations where previously at higher speeds the peculiar signal behavior was observed. The signals are likely caused by footprints of instabilities from the widening shear layer.

Probably due to the small sensor size (see Section 2) the sensor is able to capture hydrodynamic structures with a small coherence length and/or high wavenumber. It is likely that the amplitude of these small structures takes very high values which were not fully captured due to the integration effect of the sensitive surface of larger microphones. It appears that this signal cannot be fully captured because of the limitation set by the AOP of the microphones. This leads to a significant overload which can also lead to a temporary malfunction.

The question arises, why this peculiar behavior of the sensors has not been observed or reported in prior studies (see Section 1). There, implementations of signal compression could result in such events not being observed due to undetectable shaping of the signal. For the sensors used in the present study, one explanation could be that the implementation of signal compression leads to the observed peculiar behavior (nonlinear distortion or malfunction).

The findings in the previous section can be summarized into three points:

- due to the small sensor size the MEMS microphone is capable of capturing small (short coherence length and high wavenumber) hydrodynamic structures.
- These structures may sometimes exhibit a very high pressure amplitude which leads to exceeding the AOP of the MEMS microphones.
- This results in signal artifacts being imprinted onto the signal for a limited but significantly long duration and sometimes temporary malfunction.

Therefore, either sensors with a higher AOP must be used or measures are required to increases the AOP value of the employed sensors. Currently, sensors with higher AOP are not available to the authors and therefore, the application of Kapton foil for increasing the AOP value is presented in the following section.

#### 4.2.2. Surface Treatment: Application of Kapton Foil

To overcome the limitations of the MEMS microphones observed in the first tests under flow conditions, a one-sided adhesive Kapton foil of 25 μm thickness was applied in order to attenuate the pressure excitation covering all the port holes on the PCB. As a positive side-effect, this coverage provides protection to the MEMS microphones on the fuselage from humidity and dust particles.

The influence of this treatment on frequency response was measured in the anechoic chamber of the aeroacoustic laboratory of DLR Göttingen. This was performed using a rectangular plate with the surface-mounted MEMS microphone array and a surface-mounted reference condenser microphone (B&K type 4944). As a sound source, an Adam A3X speaker was used in the frequency range of 50 Hz to 60 kHz. The Kapton foil response was achieved by comparing the measurement with foil to the one without foil with respect to the reference microphone. The reference microphone was used to take into account the influence of the setup such as imperfections of the anechoic environment and differences in the excitation from the speaker. Thus, the transfer function of the Kapton foil $H_{K}$ was calculated in the frequency domain by comparing the cross-spectrum *R* between the case of MEMS microphones with Kapton applied $X_{K}$ to the case of MEMS microphones without Kapton *X*_K_ by means of a reference microphone *Y*.
(3)
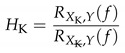

for each MEMS microphone. Here, the 24-bit GBM Viper-HDR data acquisition system was used (see Section 3.1). The measurement was performed for 60 s at a sampling frequency of 200 kHz and processed using an overlap of 50% and a fast Fourier transform block size of 5000 samples, with a Hann window, yielding 4799 averages and a narrowband frequency resolution of 40 Hz. The calculated result was then smoothed with a Savitzky-Golay filter [19] with a window length of 51 and a poly order of three.

In order to investigate the dependence on the application procedure, Kapton foil was applied a second time onto the MEMS microphone array and the measurement procedure was repeated. In the second application, a felt card was used as a tool to apply the contact pressure. It should be noted that measurement #1 was performed with the very exact Kapton foil applied to the MEMS microphone array as used in the final flight test (Section 5), without removing the foil in between.

**Figure 9:**
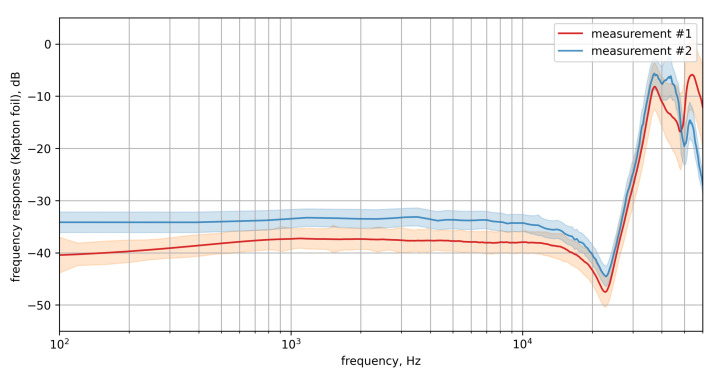
Mean frequency response of all array sensors treated with Kapton foil with standard deviation for two different applications of the coating with the Kapton foil (measurement #1: exact treatment as used in this paper). The respective standard deviation is depicted by the envelopes.

Figure 9 shows the resulting frequency response with the confidence interval for both measurements. Both results show an approximately flat attenuation of −40 dB to −38 dB (measurement #1) and −34 dB (measurement #2). After a minimum at 23 kHz, the attenuation drops to about −10 dB to −5 dB at 36 kHz. Thereafter, it further increases and decreases in the attenuation range from −10 dB to −25 dB with different courses for both measurements up to 60 kHz.

As an important outcome, the comparison of both results shows that the application of the Kapton foil can lead to different results, dependent on the method of application (usage of pressure tool, applied contact pressure). Thus, the transfer function should be measured individually for each Kapton foil application.

#### 4.2.3. Validation Measurements (TWG)

The validation measurements at increased Mach numbers were performed in the Transsonic Wind Tunnel Göttingen (DNW-TWG) facility, which is a DNW research and development facility at the DLR Göttingen site. This wind tunnel of Göttingen type has a 1.0 m by 1.0 m closed test section and can be operated for Mach numbers from of M = 0.3 to M = 2.2 under vacuumed and pressurized conditions.

**Figure 10:**
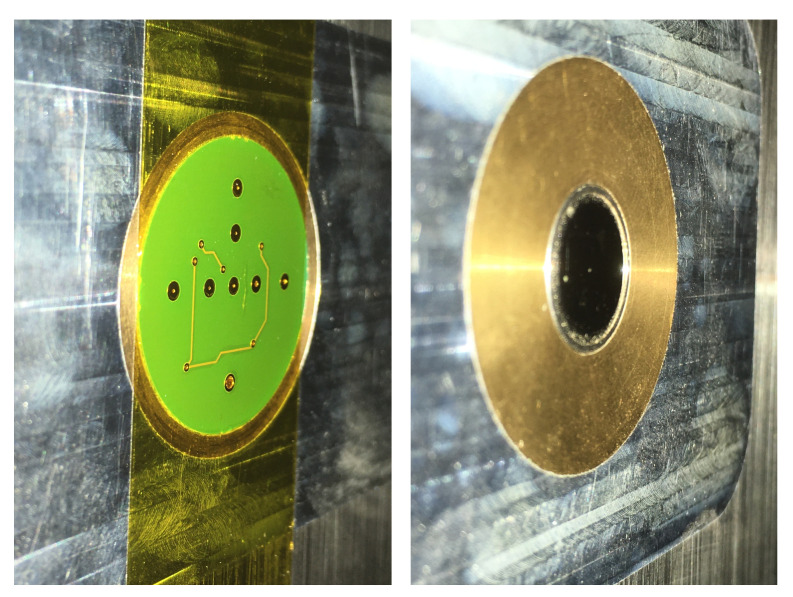
Photos of the microphones on the wind tunnel wall. Left: MEMS microphone array with Kapton foil. Right: flush mounted reference 1/2 inch microphone.

Here, embedded in another experiment, the MEMS microphones used in this paper treated with Kapton foil and a 1/2 inch B&K microphone (type 4134) were compared at a Mach number of 0.3. The microphones were flush-mounted into the wind tunnel wall as shown in Figure 10. For this measurement, the 16-bit GBM Viper-48 data acquisition system was used (see Section 3.1) using a 500 Hz high pass filter. The results were later corrected with regard to the filter response.

**Figure 11:**
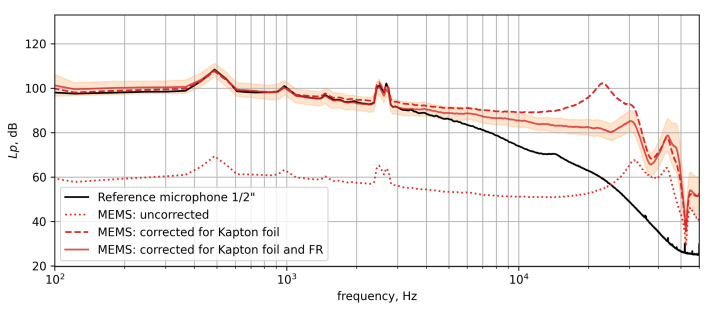
Comparison of the MEMS microphone spectrum and the reference microphone spectrum in the TWG at a Mach number of 0.3. The orange envelope illustrates the combined error margin obtained from the Kapton foil and frequency response measurements.

The spectra of a Kapton-foil-treated MEMS microphone and a reference microphone are presented in Figure 11. The black line shows the spectrum of the reference microphone. To illustrate the influence of the frequency response corrections explained in Section 4.1 (frequency response of the MEMS microphone itself) and Section 4.2.2 (Kapton foil) three different spectra are shown for the data acquired using the MEMS. The uncorrected spectrum is shown by the red dotted line, whereas the spectrum corrected for the Kapton foil is shown by the red dashed line. The fully corrected spectra are shown by the red solid line. The uncertainty range, which results from the combined standard deviations of the two correction measurements, is depicted by the orange envelope.

First, the fully corrected MEMS microphone spectrum is compared to the spectrum of the reference microphone. Second, the application of the frequency response corrections is assessed.

From 100 Hz up to a frequency of 3 kHz both spectra collapse very well, showing an slight downward slope of about 10 dB. Within this frequency range, some peaks appear at 500 Hz and 1 kHz and a double peak at about 2.5 kHz. After 3 kHz the reference microphone spectrum continues the downward slope until about 50 kHz where it transitions to a constant floor level of 25 dB at 60 kHz. The MEMS microphone spectrum, however, continues with a much less significant downward slope up to a frequency of 25 kHz. Then the spectrum decreases with superimposed ripples in the downward slope down to a level of 50 dB.

For the interpretation of the spectra, it is assumed that the broadband spectra are dominated mainly by the boundary layer noise. In the wind tunnel, no artificial sound source was present, thus only the peaks at 500 Hz and 1 kHz (wind tunnel drive, 1st and 2nd Blade Passing Frequency (BPF) order) and the peaks around 2.5 kHz are assumed to be of acoustic nature. The frequency where the strong downward slope starts (3 kHz vs. 25 kHz) is similar to the modeled attenuation for the correspondent sensor sizes in Figure 1, Section 2.1.

The estimated amplitude attenuation due to surface integration shown in Figure 1 exhibits significantly less attenuation than can be seen between the MEMS microphone signal and the reference microphone signal in Figure 11. The attenuation difference between a 1 mm MEMS microphone and a 1/2 inch microphone due to surface integration (Section 2.1) was estimated to be approximately −1 dB at 10 kHz in Figure 1. However, at 10 kHz in Figure 11, a difference in power level of approximately 10 dB is observed in the measurement. The remaining difference is due to the strong influence of a short coherence length of the TBL pressure fluctuations as discussed in Section 2.2.

For validation, the application of the frequency response corrections on the MEMS microphone is discussed. Applying the Kapton foil correction leads to an increase of the spectrum by approximately 40 dB. With the foil correction alone, the MEMS spectrum’s overall amplitude coincides well with the reference microphone below 3 kHz. At 22 kHz an additional hump is now observed which coincides with the resonance frequency of the MEMS microphones already known from the calibration in Figure 6. Applying the MEMS calibration curve to the measured spectrum removes the hump at 22 kHz and leaves a smooth spectrum up to 26 kHz. At higher frequencies, the spectrum still shows a wavy response which already is observed in the uncorrected spectrum. It can be concluded that in the frequency range greater than 26 kHz, the corrections cannot be meaningfully applied.

## 5. Application for In-Flight Measurements

### 5.1. Setup

**Figure 12:**
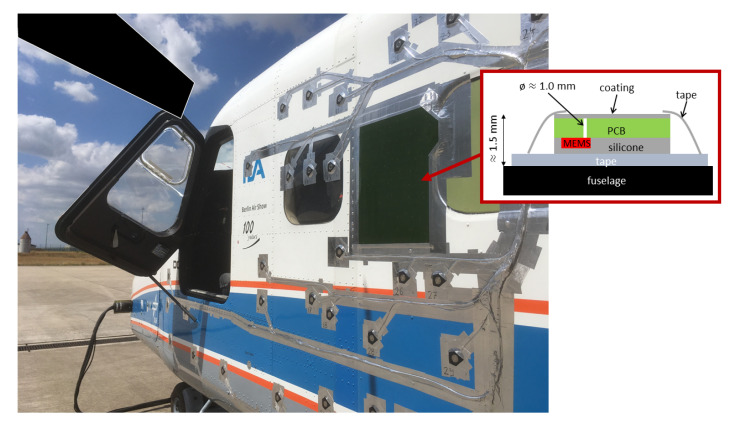
Photo of the propeller aircraft with the MEMS microphone array mounted on the fuselage and a sketch depicting the MEMS microphone array mounting.

Experiments were conducted on a DLR test carrier, the Dornier 228-101 propeller aircraft as shown in Figure 12. One window bank on the left-hand side of the aircraft was equipped with a dummy window. The dummy window allowed the passage of the cabling from the MEMS array which was attached on the fuselage close to the window and directly opposite of the propeller.

Figure 12 also shows a sketch of the MEMS microphone array mounting. The fuselage was coated with a flight-approved aluminum tape. As described in Section 3, the backside of the PCB of the MEMS microphone array was coated with silicone. Thus, the MEMS microphone array was applied via adhesion between the tape and the silicone. In order to attenuate the pressure excitation as motivated in Section 4.2.2 the MEMS microphone array was also coated with a Kapton foil. For the final fixation, the array was completely secured with the flight-approved tape around the edges.

Additionally, an array of 1/2 inch aerospace surface microphones (Brüel&Kjær type 4948) was mounted on the fuselage. Surface microphones in the vicinity of the MEMS microphone array will be used as a reference here. All signals were routed to the data acquisition system inside the aircraft. Here, the 24-bit GBM Viper-HDR data acquisition system was used (see Section 3.1) using a 500 Hz high pass filter. The results were later corrected with regard to the filter response. The microphone signals of the MEMS microphone array and the surface microphones were recorded simultaneously with a sampling frequency of 250 kHz. Spectra will be shown up to a frequency of 5 kHz. To reduce the influence of the low-frequency boundary layer noise, high-pass filter with a cutoff frequency of 500 Hz was used. The acquired data were corrected with regard to the filter response. The data were processed using an overlap of 50% and a fast Fourier transform block size of 65,536 samples, with a Hann window, yielding a narrowband frequency resolution of 3.81 Hz.

**Figure 13:**
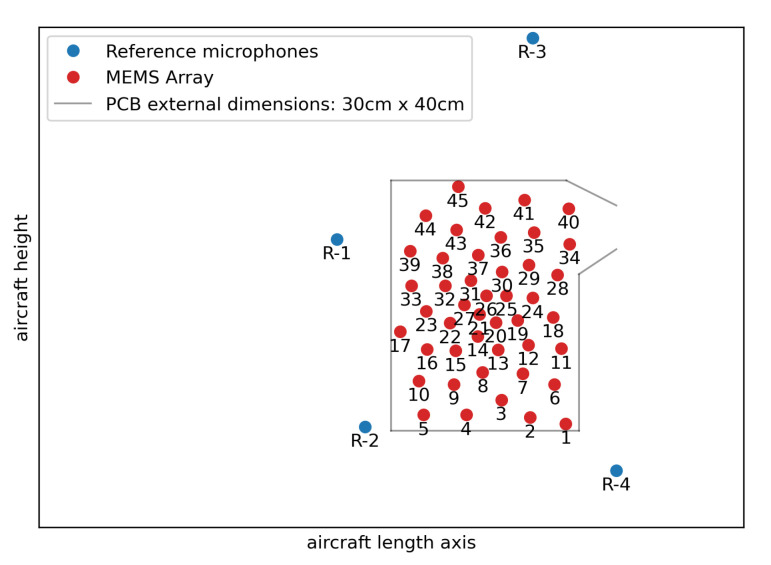
Position of all MEMS microphones (red) and the chosen reference surface microphones (blue).

Figure 13 depicts the positions of all MEMS microphones and the surface microphones chosen as a reference.

### 5.2. Results

The spectral content of the signals from both the reference microphones and the MEMS microphones in the form of an array yield valuable information about the characteristics of the surface pressure fluctuations. This information can be deduced from the autospectra and by looking at the wavenumber maps computed from the signals of the MEMS microphone array.

#### 5.2.1. Autospectra of MEMS and Reference Microphones

**Figure 14:**
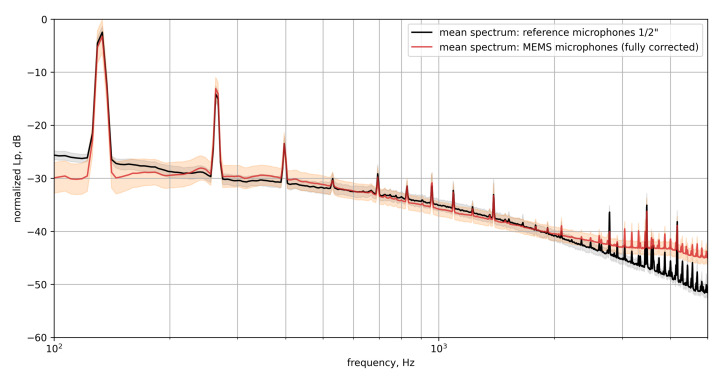
Comparison of the mean spectra of the MEMS microphones and the reference microphones with standard deviation.

Figure 14 compares the mean spectra of the reference microphones to the MEMS microphones obtained from one in-flight measurement.

In general, both spectra show a general broadband decrease over the frequency and a dominant peak at approx 133 Hz with its *n*-th order harmonics. It can be assumed, that the dominant structures are of acoustic nature. The dominant peak at 133 Hz and its *n*-th order harmonics are related to the propeller. The speed of the propeller was measured to be n≈1590 rpm. Taking into account the five propeller blades this leads to a BPF $f_{\mathrm{BPF}}\approx 26.5\;\mathrm{Hz}\times 5\approx 133\;\mathrm{Hz}$ which equals the measured peak frequency.

While the dominant tonal components (which are assumed to be of acoustic nature as they coincide with multiples of the BPF) measured with both the reference microphone and the MEMS microphone are very similar, some differences are visible for the broadband shape of the signal.

Above 2 kHz the reference microphone exhibits a stronger drop in signal compared to the MEMS microphone. This drop in signal amplitude at higher frequencies is due to the surface integration of the larger microphone modeled in Section 2.1, shown in Figure 1. Here, the 1/2 inch microphone exhibits a strong level decrease at about 2 to 3 kHz.

#### 5.2.2. Wavenumber Maps

Wavenumber maps were calculated by applying a beamforming algorithm with planar wave steering vectors without removing the diagonal elements of the cross-spectral matrix as done by Haxter and Spehr [3]. In the present case, the normalized focus grid was of size 255 by 255, extending over a normalized wavenumber range of $-10\leq k_{x,y}/k_{0}\leq 10$. A DAMAS 2.1 [20] deconvolution algorithm using $N=10^{5}$ iterations was applied to the maps for enhancement.

**Figure 15:**
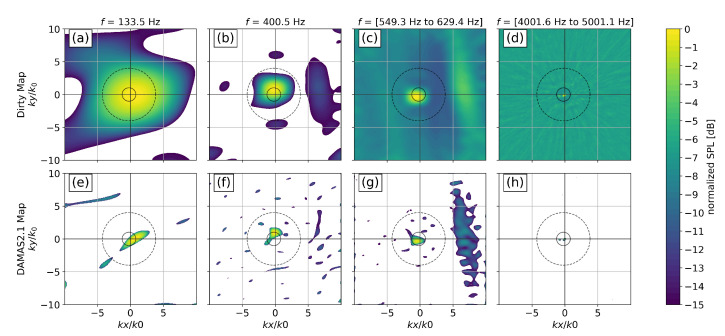
Wavenumber dirty maps and deconvolved maps at selected frequencies, plotted with a dynamic range of 15 dB. The acoustic domain is shown as a solid circle in the center of the map. The expanded integration border for separation of acoustic and other types of pressure fluctuations is shown as a dashed line surrounding the acoustic domain. (**a**)/(**e**): narrow band dirty map/deconvolved map at 133.5 Hz; (**b**)/(**f**): narrow band dirty map/deconvolved map at 400.5 Hz; (**c**)/(**g**): integrated dirty maps/deconvolved maps from 549.3 Hz to 629.4 Hz; (**d**)/(**h**): integrated dirty maps/deconvolved maps from 4001.6 Hz to 5001.1 Hz.

Resulting maps at are shown in Figure 15 for the first BPF of 133.5 Hz in Figure 15a,e, the third BPF of 400.5 Hz in Figure 15b,f, and selected broadband ranges in Figure 15c,g as well as Figure 15d,h. The top row in Figure 15 shows the direct beamformer output (or “dirty map”) without the DAMAS 2.1 enhancement algorithm applied to it. The bottom row shows the output of the DAMAS 2.1 algorithm for the respective frequency or frequency range of the map shown above. The solid circle in the center of each map represents the domain of the acoustic propagation mechanism. Sources located within this circle propagate at the speed of sound or above. The dashed circle shown is the integration map used to separate the acoustic surface pressure fluctuations from all other pressure fluctuations. To compensate for the main lobe width of sources even after a deconvolution attempt, the acoustic domain was increased to $3k_{0}$ which should incorporate the remaining imprecision in source appearance. The flight speed was sufficiently low for the convective ridge to be located reasonably far away from the acoustic domain. This gap allowed for the increase in acoustic domain threshold with no overlap of enlarged acoustic domain and convective ridge occurring.

At different frequencies, different sources can be seen to dominate the spectrum. At the first BPF at f≈133 Hz a single source is visible in the center of the map in Figure 15a,e. In the upper Dirty Map, the source is located in the center of the map but is spread out over a very large wavenumber region due to the poor resolution at low frequencies. Applying the DAMAS 2.1 deconvolution in the lower map reduces the source size, but also introduces some ghost sources at various positions in the map. However, source integration over the acoustic domain is enhanced since the size of the central source is reduced, enabling a sharper separation. Relative to the acoustic domain, the center of the dominant source appears to be located on the right-hand side of the acoustic domain, indicating a propagation direction from front to back over the array.

For the third BPF at f≈400 Hz in Figure 15b, a dominant source occurs within the acoustic domain as well. Its location relative to the acoustic domain appears to be on the upper edge of the domain, indicating a propagation at the speed of sound from the lower end of the array towards the top. Besides this acoustic source, there are four other appearances in the wavenumber map worth mentioning. The first appearance is a large elongated source on the right-hand side representing the TBL pressure fluctuations at subsonic speeds. As mentioned in Section 2.2, this convective ridge inherits its shape from the limited coherence lengths of the TBL pressure fluctuations. With the source being located to the right of the map origin and its main elongation being in $k_{y}$-direction it can be concluded that the flow is passing over the array from front to back (as expected) and that the coherence length in cross-flow direction is smaller than the coherence length in in-flow direction. Two other sources of interest are located above and below the center acoustic source at $k_{x}/k_{0}\;\approx \;0$ and $5\geq k_{y}/k_{0}\geq 6$. With the propeller passing in an upward motion on its side facing the array, the upper source location represents the direct near-field pressure fluctuation passing over the array. The lower source is possibly caused by a reflection of the near-field pressure fluctuations at the bottom side of the wing, thus propagating over the array in the opposite direction from top to bottom. It appears that the bottom source is located closer to the origin of the spectrum than the upper source, indicating that the wavenumber of the presumably reflected source is slightly smaller.

The fourth source is located towards the left of the acoustic domain, indicating an upstream propagation below the speed of sound. We could not find an explanation for this source so far. Whether or not this source is real or is just an artifact of signal processing remains an open question. Other sources in the corners of the map are likely side lobes from real sources in the map.

With DAMAS 2.1-enhancement applied to the source map at 400.5 Hz in Figure 15f, the size of the center acoustic source is significantly reduced. However, the shape of the convective ridge on the right-hand side has been dissolved. Many ghost sources have appeared over the entire map. While the two sources from the near-field of the propeller above and below the acoustic domain are still present and even have increased in relative amplitude with enhancement, their presence does not stand out any more relative to the ghost sources.

In the frequency range 549.3 Hz ≥f≥ 629.4 Hz in Figure 15c both an acoustic source in the bottom left part of the acoustic domain, as well as the convective ridge on the right-hand side, are visible. A high background noise level is present in the map. The DAMAS 2.1 enhancement is able to remove most of the background noise with the convective ridge keeping its characteristic shape. Several ghost sources are visible within the confinement of the extended acoustic domain. Regarding the near-field sources visible at lower frequency, there is only a small dot visible in the enhanced bottom map at the lower position. This however might just be a spurious processing artifact.

The last frequency range is shown in Figure 15d,h from 4001.6 Hz to 5001.1 Hz. Only two distinct acoustic sources are present: a dominant source in the center and a secondary source on the the left hand side of the acoustic domain. The remaining noise in the map exhibits a slight radial pattern centered around this acoustic source which is a characteristic feature of distinct source maps integrated over several frequencies. The image enhancement by DAMAS 2.1 is able to singularize this source, leaving only thin traces of noise behind. Remarkably, there is no convective ridge visible which is likely due to the small coherence length of TBL pressure fluctuations at these high frequencies. The array was not designed to measure this kind of pressure fluctuations and thus the convective ridge vanishes.

#### 5.2.3. Separation of Spectra

**Figure 16:**
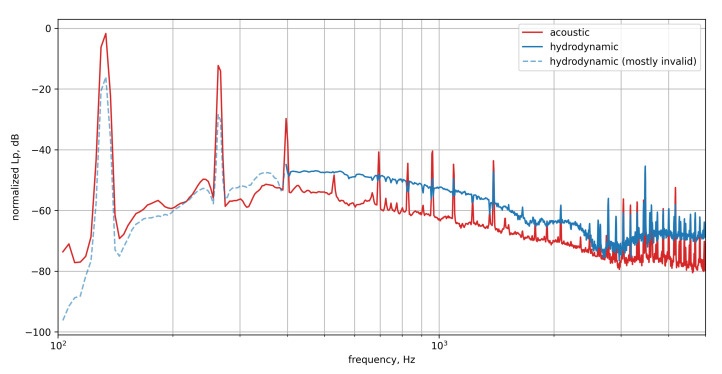
Separated spectra of acoustic pressure fluctuations and hydrodynamic pressure fluctuations. The performance of separation is best in the mid-frequency range as indicated by the solid line for the hydrodynamic pressure fluctuations.

Using the extended acoustic domain as a separation between the propagation types, the deconvolved source maps can be integrated over each separated region to obtain the separated spectra. For a noise threshold of −15 dB the separated spectra are shown in Figure 16. The spectrum obtained from integrating the extended acoustic domain is shown in red. It exhibits very distinct peaks at the BPF and higher harmonics. With increasing frequency, the broadband noise level appears to be connected to the level of hydrodynamic pressure fluctuations shown in blue. This is likely due to ghost sources resulting from incomplete source map enhancement by the DAMAS2.1 leaking into other parts of the spectrum.

The hydrodynamic spectrum is shown in blue in Figure 16 and it exhibits a rather complex appearance. At lower frequencies, indicated by the dashed blue line, a distinct drop in power is visible. This is very likely due to the turbulent structures breaking down at low frequencies as their size is limited by the boundary layer thickness. This diminishes the coherent pressure footprint and considerably reduces the signal correlation between different microphones which is required for wavenumber analysis. The acoustic peaks mostly remain unchanged as the acoustic propagation still leaves a coherent signal on all transducers in the array. The increased hydrodynamic power at the BPF is likely caused by both, the limited dynamic range of the analysis as well as near field effects as observed in Figure 15b being located outside the extended acoustic domain used for source separation.

An increased constant noise floor would likely not be reconstructed by the DAMAS 2.1 algorithm as such but rather be approximated by distributed small sources that would explain the noise. Additionally, the likelihood of phase mismatches between measurement and Green’s function is increased at higher frequencies which could explain a drop in efficiency of the enhancement algorithm. The close resemblance of the acoustic spectrum by the hydrodynamic spectrum at high frequencies is again likely due to the limited dynamic range of the analysis.

In the mid-frequency range from ≈400 Hz to ≈2.2 kHz the hydrodynamic spectrum exhibits a slow decrease of fluctuation power with intermediate peaks at the higher harmonics of the BPF which are likely caused by the near-field of the propeller. At slightly higher frequencies, the influence of the peaks at BPF increasingly dominates the hydrodynamic spectrum. Whether or not this is a real phenomenon remains uncertain at this time.

Overall, the wavenumber spectra provide valuable insights into the origins and characteristics of the surface pressure fluctuations when measured by an array of surface-mounted sensors.

## 6. Summary

The paper describes the process of preparing MEMS microphones within a flexible circuit board array for the measurement of unsteady pressure fluctuations on an airplane fuselage during flight tests.

The MEMS sensors have a small sensitive surface area which allowed them to capture the presence of high-energy small-scale structures within the turbulent boundary layer when pre-testing them in a low-speed wind tunnel environment. Although the relationship between the sensitive surface area size of the sensor and an increasingly high measured amplitude is known, it can easily be overlooked if such high amplitudes are not expected. As measurements nowadays are performed more frequently using small-scale MEMS sensors, higher measured amplitudes are bound to occur more frequently as well.

With the high measured amplitudes resulting from the matched size ratio of the turbulent boundary layer pressure fluctuations to the sensitive surface area of the sensor, it is possible to elicit nonlinear behavior in the response of the MEMS sensors due to them temporarily exceeding the upper limit of their dynamic range.

Some makes of MEMS sensors respond to exceeding this limit by either clipping or compressing the signal, which may cause an inaccurate underestimation of the amplitude of the small-scale hydrodynamic pressure fluctuations. If compression is used by the internal signal conditioning of the sensor, such a distortion may go unnoticed.

To overcome these limitations of current MEMS microphones and be able to apply them in flight tests, the sensitivity of the MEMS array was decreased by the application of Kapton foil of 25 μm thickness. The damping resulting from the applied foil was measured and revealed a mean insertion loss of 34 to 40 dB with a variation of ±3 dB between single microphones in the frequency range of interest.

After a low-speed test, the Kapton-covered MEMS were reviewed further in a high-speed wind tunnel test. The test was aimed at comparing the signals from a reference 1/2 inch microphone to the signal from the MEMS microphones with frequency response correction and Kapton foil response correction applied. The analysis of the high-speed lead to the conclusion that the application of both frequency responses to the MEMS microphone signals leads to a very good agreement with the reference signals with expected differences due to sensitive surface size.

Finally, flight tests were conducted on the DLR Dornier 228 propeller aircraft with the MEMS microphone array mounted on the left-hand side of the fuselage, opposing the left-hand-side propeller. The comparison with 1/2 inch microphones mounted in the vicinity of the MEMS microphone array shows a good general signal agreement in the expected frequency range when applying the appropriate frequency corrections. Apart from the good general agreement, some differences were visible as well. The mean broadband spectra of the MEMS microphones revealed more fluctuating energy especially in the range above 2 kHz. Using a wavenumber analysis it was determined that these increased levels were caused by hydrodynamic pressure fluctuations.

The results indicate that the Kapton foil covering the MEMS microphones was able to damp the pressure fluctuations as expected, while still preserving the critical phase information required for wavenumber analysis.

A great advantage of MEMS microphones lies in their small size and the possibility to arrange them closely-spaced and flush-mounted in a very thin array that can be applied to follow the shape of the underlying fuselage structure. The close spacing and small sensitive surface area of the sensors allows for analyzing the wavenumber spectra of both, the acoustic pressure fluctuations as well as the overlaying turbulent boundary layer. The results of the wavenumber analysis from the flight test measurements reveal the presence of both the acoustic pressure fluctuations induced by propeller and engine and the convective ridge of the hydrodynamic pressure fluctuations at different relative amplitudes depending on frequency.

It can be concluded that MEMS microphones are an inexpensive alternative to conventional microphones even at challenging experimental conditions during flight tests. Due to their small size the MEMS exhibit an increased potential for spatially high-resolved measurements which larger sensors are not capable of.

## Abbreviations

The following abbreviations are used in this manuscript:

A/D	Analog-to-digital converter
AOP	Acoustic Overload Point
BPF	Blade Passing Frequency
DAMAS	Deconvolution Approach for the Mapping of Acoustic Sources
DLR	Deutsches Zentrum für Luft-und Raumfahrt (German Aerospace Center)
DNW	Deutsch-Niederländische Windkanäle (German-Dutch Wind Tunnels)
FR	Frequency response
MEMS	Micro Mechanical System
PCB	Printed Circuit Board
SNR	Signal-to-Noise Ratio
THD+N	Total Harmonic Distortion + Noise
TWG	Transonic wind tunnel facility
